# Algorithms and instrumentation for rapid spatial frequency domain fluorescence diffuse optical imaging

**DOI:** 10.1117/1.JBO.27.11.116002

**Published:** 2022-11-08

**Authors:** Sang Hoon Chong, Vadim A. Markel, Ashwin B. Parthasarathy, Yi Hong Ong, Kenneth Abramson, Frank A. Moscatelli, Arjun G. Yodh

**Affiliations:** a University of Pennsylvania, Department of Physics and Astronomy, Philadelphia, Pennsylvania, United States; b University of Pennsylvania, Department of Radiology, Philadelphia, Pennsylvania, United States; c University of South Florida, Department of Electrical Engineering, Tampa, Florida, United States; d New York University, Department of Physics, New York, United States

**Keywords:** structured illumination, diffuse optical tomography, fluorescence, image-guided surgery

## Abstract

**Significance:**

Rapid estimation of the depth and margins of fluorescence targets buried below the tissue surface could improve upon current image-guided surgery techniques for tumor resection.

**Aim:**

We describe algorithms and instrumentation that permit rapid estimation of the depth and transverse margins of fluorescence target(s) in turbid media; the work aims to introduce, experimentally demonstrate, and characterize the methodology.

**Approach:**

Spatial frequency domain fluorescence diffuse optical tomography (SFD-FDOT) technique is adapted for rapid and computationally inexpensive estimation of fluorophore target depth and lateral margins. The algorithm utilizes the variation of diffuse fluorescence intensity with respect to spatial-modulation-frequency to compute target depth. The lateral margins are determined via analytical inversion of the data using depth information obtained from the first step. We characterize method performance using fluorescent contrast targets embedded in tissue-simulating phantoms.

**Results:**

Single and multiple targets with significant lateral size were imaged at varying depths as deep as 1 cm. Phantom data analysis showed good depth-sensitivity, and the reconstructed transverse margins were mostly within ∼30% error from true margins.

**Conclusions:**

The study suggests that the rapid SFD-FDOT approach could be useful in resection surgery and, more broadly, as a first step in more rigorous SFD-FDOT reconstructions. The experiments permit evaluation of current limitations.

## 1 Introduction

Surgical guidance based on fluorescence imaging is increasingly being explored as a means to aid tumor resection ^1^^,^^2^ in neuro-^3^^–^^6^ and thoracic^7^^,^^8^ surgeries. The primary goal of surgical resection is to excise all cancerous tissue since leftover residual tumor cells can lead to local cancer regrowth and poor clinical outcomes. Currently, surgeons rely on visual inspection, palpation, or intraoperative pathology to identify tumor boundaries and residual tumor cells; however, the specificity and spatial resolution of these methods are poor. Fluorescence imaging addresses these limitations by providing spatially resolved, real-time contrast between tumorous and healthy tissues. The technique relies on exogenous contrast agents that accumulate preferentially in tumors, such as the widely-used and FDA-approved fluorophore indocyanine green (ICG) and some newer cell-specific fluorophores.^9^ Fluorescence from these contrast agents helps demarcate boundaries between cancerous and healthy tissue.

Currently, fluorescence image guidance for surgical tumor resection is accomplished with commercial and research-grade imaging systems^10^^,^^11^ that utilize epi-illumination with near-infrared (NIR) light and that image fluorescence from the surgical scene with wide-field camera-based detectors. In practice, two-dimensional (2D) fluorescence images from the tissue (including the tumor regions) are overlaid on a corresponding 2D bright-field image, thereby enabling surgeons to ascertain the transverse boundaries of the tumor. Since preserving normal tissue is critical for organs such as brain, accurate detection of tumor margins is important. For example, in the case of neurosurgery for tumor resection, accurate geometric information about the location and margins of tumors below the surface could be utilized for decisions concerning surgical entry point on the tissue surface and concerning the path of minimal damage to the healthy tissue. In this regard, current fluorescence imaging suffers from technical limitations. First, 2D fluorescence imaging is most accurate only for tumors at or near the tissue surface, and it cannot determine tumor depth. Second, diffusion of the fluorescent light from the tumor can make the image margins appear larger than the actual tumor margins;^12^ this effect is more pronounced for subsurface tumors, i.e., the overestimation of tumor transverse margins by 2D-projection fluorescence imaging increases significantly with tumor depth. Improved localization of the tumorous tissues in three-dimensions (3D) is, therefore, desirable for better surgical planning and outcomes.

In this work, to improve localization of tumors during surgery, we adapt a parallel set of diffuse optical fluorescence imaging advances that predate the current commercial approaches for fluorescence image guidance.^13^^–^^29^ While these early advances have rarely been deployed clinically, they hold potential for wide-field, noncontact fluorescence imaging with real-time processing. Recent advances based on the more complex time-of-flight^28^ and neural network^27^^,^^29^ DOT instrumentation and approach have exhibited excellent reconstruction speed and spatial resolution, though these techniques still need further validation to be applicable in OR. Another advance in clinical imaging utilizes two different wavelengths of fluorescent emission^30^ to derive information about the tumor depth;^31^ however, information about the tumor lateral margins is not obtained by this method. The other technique, spatial frequency domain fluorescence diffuse optical tomography (SFD-FDOT), was used to produce 3D images of small point-like fluorophore targets^19^ in phantom experiment and in mouse models. SFD-FDOT illuminates tissue with continuous-wave, wide-field intensity patterns, which are sinusoidally modulated at different spatial frequencies. With this scheme, light penetration depth is controlled by the spatial frequency, and the wide-field image data collected at different spatial frequencies facilitates 3D reconstruction of the fluorophore concentration. In principle, the SFD-FDOT method allows one to acquire 3D information about the tumor depth and lateral margins. However, prior implementations of SFD-FDOT were limited to point-like fluorophore targets (diameter of 2–3 mm) with limited depth sensitivity.^19^^,^^24^

Here we report on an instrument and a new rapid reconstruction algorithm for SFD-FDOT. Our analysis builds on the spatial frequency domain approach and introduces a rapid and computationally inexpensive two-step reconstruction algorithm. In the first step, we use the variation of reflected diffuse fluorescence intensity with respect to the spatial modulation frequency of incident light to estimate the tumor depth. Then, using the tumor depth determined in the first step, we determine the lateral margins of the fluorophore concentration in the target plane by rapid analytical data inversion. We report the principles and details of this methodology and we exhibit the results from a series of SFD-FDOT phantom experiments, which characterize the depth accuracy and lateral spatial resolution of the proposed method. The findings reported below suggest that the methodology could be useful clinically by enabling rapid tumor localization and margin assessment. Additionally, it can be used to provide constraints for more rigorous or comprehensive fluorescence tomography.

## 2 Methods

### 2.1 Instrumentation

**Fig. 1:**
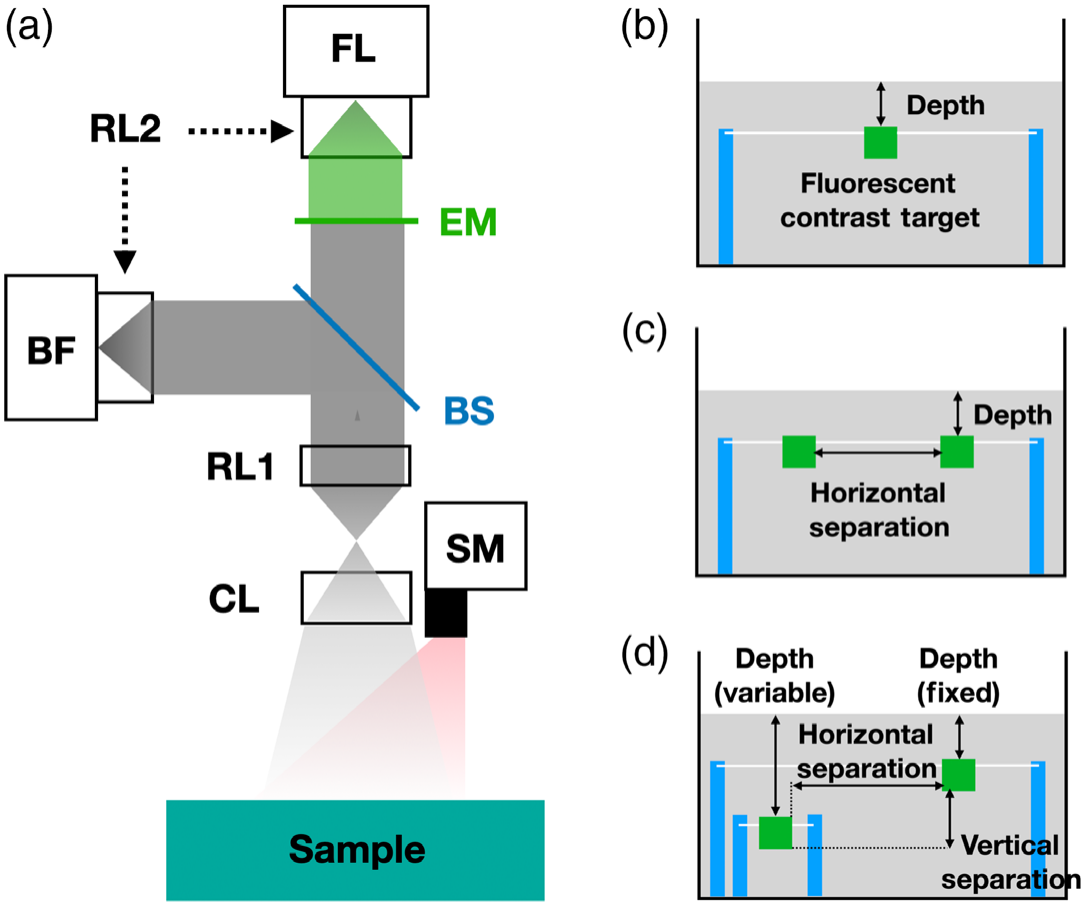
(a) Schematics of the dual-camera imaging system. SM, Spatial modulator of the excitation light; CL, collection lens; RL, relay lens; BS, 10:90 beam-splitter; BF, bright-field camera; EM, emission filter; FL, fluorescence camera. (b) Tissue-simulating liquid phantom and a 3D printed hollow cube ($1\;{\mathrm{cm}}^{3}$) containing a mixture of ICG and a scattering solution. For the single-target experiments, the cube was suspended in a tissue-simulating liquid phantom using two posts (blue), and the target depth was adjusted by varying the surface level of the background phantom. (c) For one set of two-target experiments, two identical targets were placed at the same depth below the surface; the transverse distance between the targets was controlled, and the target depth was adjusted as in (b). (d) For the second set of two-target experiments, two targets were separated both horizontally and vertically. The right target was held at 2 mm below the surface, and the depth of the left target was adjusted.

We have constructed a wide-field imaging system with a digital micro-mirror device for illuminating the tissue at different spatial frequencies and a spectrally-separated dual camera detection system, which records simultaneously bright-field reflectance images at the excitation wavelength of 808 nm and the fluorescence images at the emission wavelength of 850 nm. The setup is illustrated schematically in Fig. 1(a). The combined illumination and imaging system was mounted on a translation stage (MN10-015-E01-13, Velmex Inc., Bloomfield, New York) and positioned above the sample with the object distance of 32 cm. The Illumination was provided by a digital light projector (FC E4500 MKII, EKB Technologies Ltd. Bat-Yam Israel). Its intensity on the sample surface was $∼0.3\;\mathrm{mW}/{\mathrm{cm}}^{2}$ for uniform (spatially unmodulated) illumination, well below the American National Standards Institute (ANSI) limit for tissue damage. A digital micromirror device was electronically controlled to produce spatially-modulated (at different frequencies) illumination of the sample surface with wide-field light at the wavelength of 808 nm. The reflected light at the excitation and fluorescent wavelengths was directed to both camera channels and was further spectrally filtered to record the fluorescence image. The images recorded in the fluorescence camera are used for reconstruction of the depth and the transverse margin of the fluorescent inclusions.

### 2.2 Experimental Procedure

The background tissue was simulated using a mixture of intralipid (Intralipid 20%, Fresenius Kabi, Pune, Maharashtra, India), nigrosin (Acid Black, MP Biomedicals, Santa Ana, California), and water. The background has wavelength-dependent absorption coefficient $\mu_{a}$ and reduced scattering coefficient $\mu_{s}^{\prime }$. In choosing optical properties, we adopted generic tissue (breast, muscle, and brain) scattering coefficients, and we chose an absorption coefficient similar to breast tissue. Respectively, these coefficients are 0.004 and $0.8\;{\mathrm{mm}}^{-1}$ at the excitation wavelength of 808 nm, and 0.006 and $0.76\;{\mathrm{mm}}^{-1}$ at the emission wavelength of 850 nm. The hollow cube was 3D printed with inner side length of 10 mm and wall thickness of 1 mm (VeroWhitePlus, Stratasys Direct Manufacturing, Valencia, California). It was employed to simulate the fluorescent target(s); the cube was filled with a mixture of intralipid containing dissolved ICG powder (IC-GREEN, Akorn Inc., Lake Forest, Illinois). 6.5  μM of ICG concentration was chosen to maximize fluorescence yield. The absorption property of background tissue and the ICG concentration were both selected to accommodate our very low-intensity excitation light. The fluorescent contrast cubes were suspended in the liquid phantom tank using fishing lines and posts, as shown in Fig. 1 [Panels (b), (c), and (d)]. The depths of the targets were controlled by lowering or raising the surface of the background liquid phantom in the single-target experiments [Fig. 1(b)]. The image was recorded with 12-bit dynamic range, and the exposure time was increased until the maximum count of a pixel reached approximately 4000 for unmodulated incident light (setting it above 4000 occasionally led to saturation of a few of the sensors.) The fluorescence camera exposure time was adjusted from 800 ms for the 2 mm depth of the target to 5 s for the 10 mm depth to account for the difference in detected intensity. The exposure time of the bright-field camera was about 15 ms. Accordingly, the full acquisition time for the deepest target was four times longer than that of the shallowest target. Fluorescence emission data for single-target phantoms were acquired for target depths of 2, 4, 6, 8, and 10 mm.

For two-target phantoms, two different sets of experiments were performed. In one set, two identical targets were placed at the same depth. This depth of the two targets, and the lateral separation between the targets, were varied as shown in Fig. 1(c). In a second set of two-target phantom experiments, the targets were laterally separated by either 20 or 40 mm. The depth of one target was fixed at 2 mm, whereas the depth of the other target was varied from 4 to 8 mm [Fig. 1(d)].

In all experiments, the data acquisition time for the fluorescence associated with the shallowest target (2 mm depth) was ∼2  min. The acquisition time for fluorescence associated with the deepest target (10 mm depth) was ∼8  min.

### 2.3 Data Processing and Analysis

Theory of the spatial frequency-domain imaging has been exposed in Refs. 19 and 32. For clarity, we provide here a concise description of the relevant ideas, largely following Ref. 19. The symbols and corresponding units for all parameters and variables are summarized in Table 1.

**Table 1:** Descriptions of parameters and variables.

Symbol	Description
ρ	2D Cartesian vector position coordinate in the (x,y) plane (mm)
z	Cartesian coordinate of the depth of the target top-surface (mm)
k	Wave number of the spatially-modulated illumination (${\mathrm{mm}}^{-1}$)
f	Spatial frequency of the spatially modulated illumination light (${\mathrm{mm}}^{-1}$)
θ	Phase offset of the spatially modulated illumination light (rad)
q	2D Fourier variable reciprocal ρ
σ	Regularization parameter for low-pass filtering
γ	2D Fourier-transform of the two-point Green’s function
C	Concentration of ICG (μM)
$\hat{C}$	2D Fourier-transform of the ICG concentration
ε	Extinction coefficient of ICG at 808 nm (${\mathrm{mm}}^{-1}\cdot \mu M^{-1}$)
η	Quantum efficiency of ICG at 808 and 850 nm (assumed to be similar at these wavelengths)
S	Light illumination intensity on the surface of the liquid phantom ($\mathrm{mW}/{\mathrm{mm}}^{2}$)
Φ	Emission fluence rate in real space representation
ϕ	2D Fourier-transform of Φ
ℓ	Extrapolation distance (mm)
D	Light diffusion coefficient (${\mathrm{mm}}^{2}/s$)

**Fig. 2:**
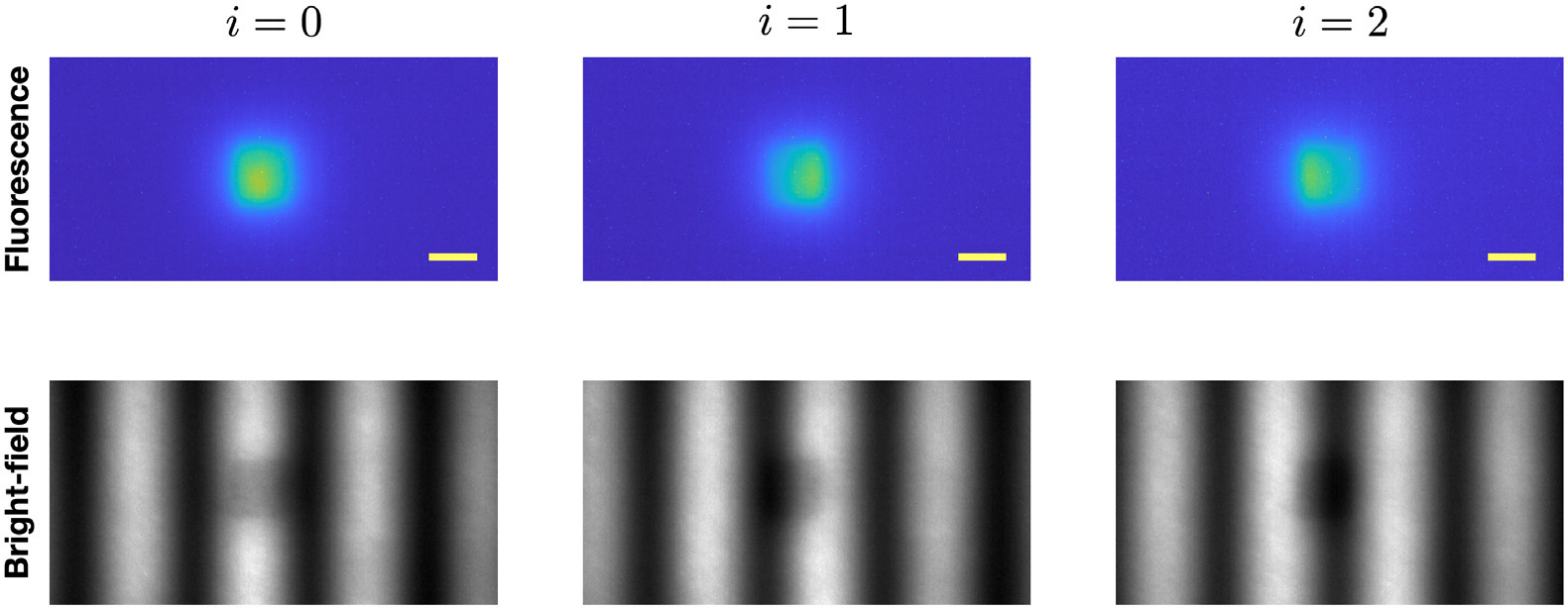
Example of the measurement in case of single target located at the depth of 2 mm ($f=0.04\;{\mathrm{mm}}^{-1}$). Scale bar indicates 10 mm.

First, we describe the illumination scheme. The sample surface is illuminated by a sinusoidally-modulated intensity pattern of the form

$$S_{i}(\rho_{d};k)=\frac{S_{o}}{2}[1+\cos(k\cdot \rho_{d}+\theta_{i})],\;i=0,1,2,$$ (1)

where $\rho_{d}=(x_{d},y_{d})$, where $x_{d}$ and $y_{d}$ are Cartesian coordinates in the surface of the sample (assumed to be a plane). The quantities $S_{o}$ and $\theta_{i}$ represent the incident intensity and the spatial phase shift of the illumination pattern. In the experimental procedure, we use $\theta_{0}=0$, $\theta_{1}=2\pi /3$, and $\theta_{2}=4\pi /3$ (this choice of phases will be explained below). In principle, it is possible to use general 2D modulation wave vectors k. However, in our experiments, we modulated the incident light only along the x-axis so that k=(k,0), where the spatial modulation wave number is k=2πf, and f is the spatial frequency of the modulating sinusoid. This restriction of the modulation wave vector is sufficient for reconstructions. Use of other modulation directions can enhance the method but requires larger data acquisition times. In the experiments, we typically employed 31 different spatial frequencies f varying from 0 to $0.15\;{\mathrm{mm}}^{-1}$. Note that, in practice, small discrepancies arise between the set of intended and actual values of f on the phantom surface due to small deviations in the estimated distance between the sample surface and the collection lens. We determine the actual values of f by analyzing the bright-field image, and we use these measured values of f in the reconstructions. In Fig. 2, examples of excitation light reflection (bright-field) images and corresponding fluorescence images are presented.

For each illumination pattern $S_{i}(\rho_{d};k)$, a corresponding fluorescent emission image $\Phi_{i}(\rho_{d};k)$ was recorded (the top row of Fig. 2). The images $\Phi_{0}$, $\Phi_{1}$, and $\Phi_{2}$ were then combined to obtain the complex fluorescent emission signal, $\Phi (\rho_{d};k)$ according to

$$\Phi (\rho_{d};k)=\frac{1}{3}[2\Phi_{0}(\rho_{d};k)-\Phi_{1}(\rho_{d};k)-\Phi_{2}(\rho_{d};k)]+\frac{i}{\sqrt{3}}[\Phi_{2}(\rho_{d};k)-\Phi_{1}(\rho_{d};k)].$$ (2)

This combination is needed for our analysis. Essentially, it allows us to remove the constant background in illumination and access the information of amplitude and phase equivalent to that provided by illuminating the medium with both sine and cosine functions in the spatial modulation pattern. We further define the 2D Fourier transform of $\Phi (\rho_{d};k)$ with respect to $q_{d}$, viz

Φ^(qd;k)=∫Φ(ρd;k)e−iqd·ρdd2ρd. (3)

Image reconstruction relies on an integral relation between Φ^(qd;k) and the heterogeneous fluorophore concentration in the sample, assuming uniform background optical properties and first-order approximation for diffuse wave propagation, which linearizes the inverse problem. The relation is of the form

Φ^(qd;k)=εη∫0∞γ(qd,z;k)C^(qd−k,z)dz, (4)

where ε is the fluorophore extinction coefficient, η is the quantum efficiency, and C^(q,z) is the depth-dependent (where depth corresponds to the coordinate z) 2D Fourier transform of the fluorophore concentration.^19^ Another important term in the integrand of Eq. (4) is the two-point Green’s function kernel, $\gamma (q_{d},z;k)$. This kernel is critical for the proposed rapid algorithm. In the semi-infinite geometry (which we assume for our problem), it has the following form:^19^^,^^33^

$$\gamma (q_{d},z;k)=\frac{\ell_{\mathrm{ex}}\ell_{\mathrm{em}}}{D_{\mathrm{ex}}D_{\mathrm{em}}}\frac{e^{-[Q_{\mathrm{ex}}(k)+Q_{\mathrm{em}}(q_{d})]z}}{\left[Q_{\mathrm{ex}}(k)\ell_{\mathrm{ex}}+1][Q_{\mathrm{em}}(q_{d})\ell_{\mathrm{em}}+1\right]}.$$ (5)

Here the subscripts “ex” and “em” indicate, respectively, quantities associated with excitation and emitted (fluorescent) light; $\ell_{\mathrm{ex}}$ and $\ell_{\mathrm{em}}$ are the extrapolation distances, and $D_{\mathrm{ex}}$ and $D_{\mathrm{em}}$ are the light diffusion coefficients. The depth penetration factor is given as

$$Q(q)=\sqrt{3\mu_{a}(\mu_{a}+\mu_{s}^{\prime })+{\left|q\right|}^{2}}.$$ (6)

Note that $Q_{\mathrm{ex}}$ and $Q_{\mathrm{em}}$ in Eq. (5) are defined by Eq. (6) but utilize $\mu_{a}$ and $\mu_{s}^{\prime }$ of the background medium at the excitation and emission wavelengths, respectively. Importantly, the kernel [Eq. (5)] decreases exponentially with the depth z. Finally, Eqs. (3)–(5) are valid for any (wave) 2D vectors of the structured illumination, k. However, for the reconstructions in this work, we have utilized samples of k taken along the x-axis so that k=(k,0).

The first step in the reconstruction algorithm is to estimate the target depth. To this end, we utilize a simplification of Eq. (4). Specifically, we assume that the fluorophores are localized at the depth of the top surface of each target, which we refer to as $z_{\text{target}}$, so that C^(qd−k,z)=C^(qd−k)δ(z−ztarget). In this case, Eq. (4) simplifies to the following form (with $q=q_{d}-k$):

Φ^(q+k;k)=εηγ(q+k,ztarget;k)C^(q). (7)

This equation can be rearranged to relate the z-dependent kernel to the measured fluorescence emission and to the fluorophore concentration distribution. Note that, even if the actual fluorophore distribution is more widely spread in the z-direction, our method will derive a fairly good estimate of the depth. Specifically, with the assumptions outlined above, and with q=0, it is straightforward to show that, for each spatial modulation wave vector k, we have

$$y(k)=A\;\exp(-z_{\text{target}}x(k))+y_{o},$$ (8a)

where

$$x(k)=Q_{\mathrm{ex}}(k)+Q_{\mathrm{em}}(k),$$ (8b)

y(k)=Φ^(k;k)[Qex(k)ℓex+1][Qem(k)ℓem+1]. (8c)

The dependent variable, y(k), is the normalized emission response at the modulation wave vector k. The independent variable, x(k), can be measured or estimated; it is, essentially, twice the reciprocal of the penetration depth, which depends on the spatial modulation frequency. The parameter $A=\varepsilon \eta (\frac{\ell_{\mathrm{ex}}\ell_{\mathrm{em}}}{D_{\mathrm{ex}}D_{\mathrm{em}}})\hat{C}(0)$ depends on the parameters of the background medium and the fluorophores, some of which may not be directly known or measured, but it is independent of the modulation wave vector k. In a data set obtained by varying k, we can view A as a constant and as an adjustable parameter. Further, $y_{o}$ is an extra adjustable parameter added to account for the noise floor in the data since the fluorescent signal does not go to zero when |k|→∞ (as predicted theoretically), perhaps as a result of background fluorescence and other noise, as well as imprecision in the theoretical model. Note that the adjustable parameters A and $y_{o}$ are expected to be different in different data sets, but in each deta set they are independent of k. We can now find $z_{\text{target}}$ by nonlinear fitting of the theoretical Eq. (8a) to the data in which A, $z_{\mathrm{target}}$ and $y_{o}$ are viewed as k-independent adjustable parameters.

Once the depth of the target is determined, we can compute the lateral margins of the fluorophore concentration in the plane. Rearranging Eq. (7) for C^(q) and taking the inverse 2D Fourier transform, we obtain a simple reconstruction formula for the transverse distribution of the fluorophore target in the plane $z=z_{\text{target}}$. The obtained relation is parameterized by k and, theoretically, any value of k can be used to obtain the transverse distribution of the fluorophore. In the reconstructions, we have used the k=0 for this purpose because the signal-to-noise ratio in the fluorescence images is best for unmodulated incident light. Therefore, we use the reconstruction formula

C(ρ,z=ztarget)=1εη∫Φ^(q;k=0)γ(q,ztarget;k=0)e−iq·ρfσ(q)d2q(2π)2, (9)

where

$$f_{\sigma }(q)=\frac{1}{2\pi \sigma^{2}}\;\exp(-\frac{q^{2}}{2\sigma^{2}})$$ (10)

is a Gaussian low-pass filtering kernel. We apply the filter to ameliorate the effects of noise and model imprecisions that render the integrand in Eq. (9) unreliable at high values of q. Since the denominator of the integrand decreases exponentially with q [see Eq. (5)], the contribution of noise to the image is amplified when large values of q are used in a numerical reconstruction. The Gaussian filter enables us to regularize Eq. (9). The regularization parameter σ depends on the level of noise and must be determined for each data set separately. However, the reconstruction is so fast that it is possible to generate many images in real time while tuning σ. In this study, we adopt a straightforward approach to find the optimum σ, i.e., σ is incremented from 0. At each value, the statistics of the noise is computed until the weak positivity constraint is met. This process is described in detail in Sec. 3.1.

### 2.4 Nonlinear Fitting

Prior to fitting, the data were normalized to the maximum value. That is, both sides of Eq. (8a) were divided by y(k=0). This allowed us to compare errors of the fit for different data sets quantitatively. We then obtained an estimate of the fluorophore target depth $z_{\text{target}}$ from the best fit of the theoretical Eq. (8a) to the data points [x(k),y(k)/y(0)]. We removed the first data point (with the smallest |k|) from the fitting procedure for the reasons explained in Sec. 3.1. For the fitting, we used a nonlinear regression model algorithm, fitnlm (MATLAB 2022a, The Mathworks Inc., Natick, Massachusetts), and chose the initial values for A, $z_{\text{target}}$, and $y_{o}$ to be 5.0, 1.0, and 0.1, respectively. For verification, we also used the fitting function of Gnuplot. Very similar results were obtained (these data are not shown below).

## 3 Results

### 3.1 Single-Target Experiments

**Fig. 3:**
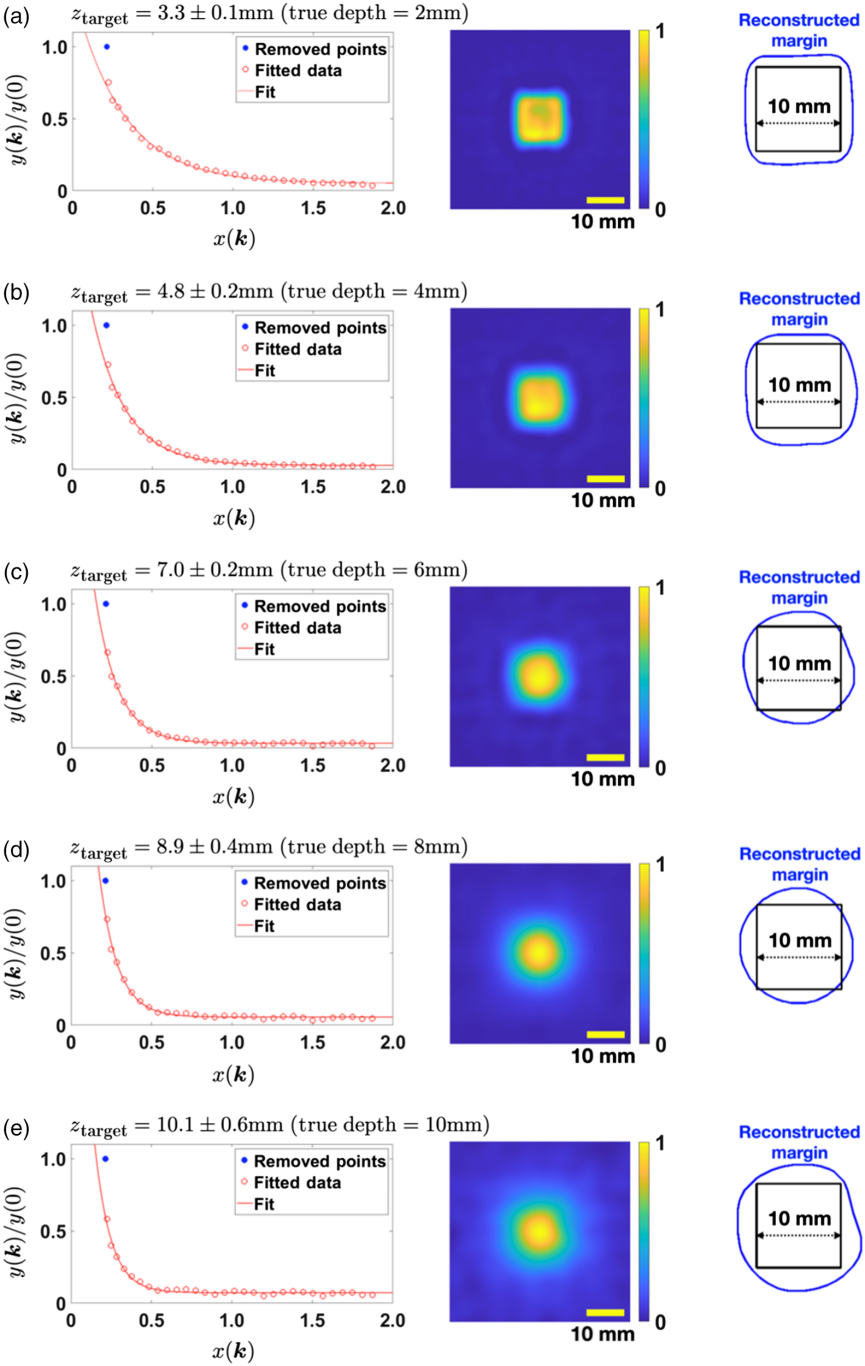
Single target experiment results. The target depth is (a) 2 mm, (b) 4 mm, (c) 6 mm, (d) 8 mm, and (e) 10 mm. The left column shows the exponential fitting of the normalized data used to estimate the target depth. The estimated depth is marked on top of the plot along with its standard deviation. The middle column shows the reconstructed transverse images. The right column shows the transverse margin from the image and the true margin. The yellow scale bar corresponds to 10 mm.

The depth of the fluorescence contrast target was varied from 2 to 10 mm, and the corresponding exponential fitting results are displayed in the left column of Fig. 3. Note that, as mentioned above, the first data point corresponding to k=0 was not used for fittings. If used, this data point can skew the decay constant to larger values and increase the fitting errors significantly. Removal of this point can be justified on grounds that the fluorescent targets employed in our experiments are not infinitely thin layers but cubes and therefore have finite depth of 10 mm. Owing to the high absorption coefficient of ICG and the small Stokes shift, we perform the reconstructions under the assumption that the fluorescent signal comes predominantly from the top faces of the cube. This assumption, however, disregards penetration of the incident photons in the interior of the fluorescent targets. As a result of this effect, the inverse problem of reconstructing the ICG concentration is nonlinear, and replacing it with a linearized problem, as is done in our theoretical analysis, is an approximation. The quality of this approximation is expected to be especially poor at k=0, which explains the anomalous position of the respective data point in Fig. 3. At higher values of k, the effects of nonlinearity appear to be weaker and can be neglected. However, a more precise corroboration of this picture will require additional (future) experiments with smaller fluorescent targets or smaller ICG concentration; if successful, inclusion of the k=0 (and other data points with relatively small values of k) will be possible and is expected to increase the dynamic range of measurements, which is useful for more accurate depth estimation.

**Fig. 4:**
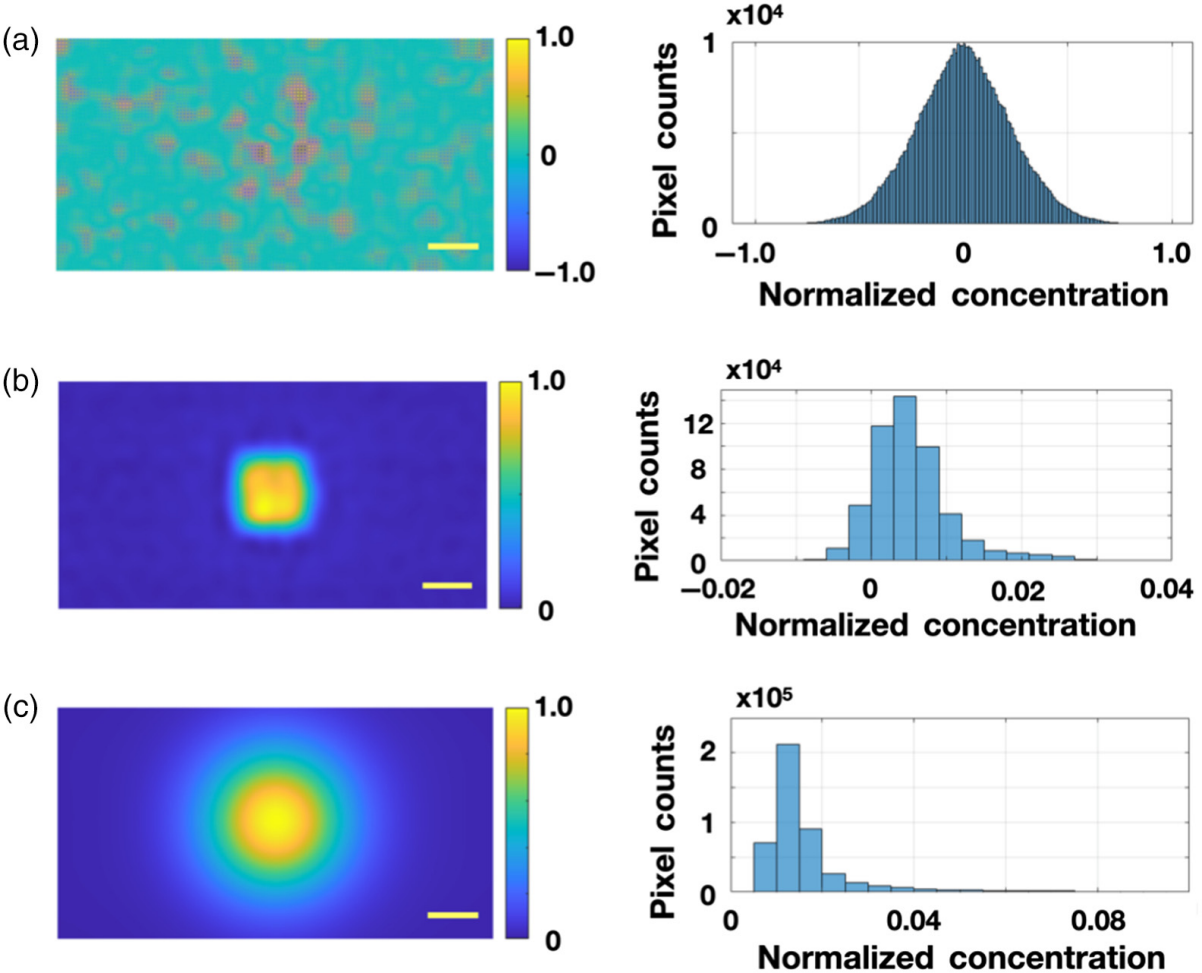
Transverse image of the fluorescent inclusion at 4 mm depth with different values of σ using the same data as in Fig. 3(b). (a) The top and bottom rows show the cases of weak regularization corresponding to $\sigma =3.77\;{\mathrm{mm}}^{-1}$ and (b) strong regularization corresponding to $\sigma =0.11\;{\mathrm{mm}}^{-1}$, The middle row was obtained using the optimal regularization (as defined in the text) with $\sigma =0.46\;{\mathrm{mm}}^{-1}$. In the optimized images, the number of pixels with negative values is relatively small (<16% of all pixels). The left column shows the reconstructed images obtained from Eq. (9) and the right column provides normalized concentration distribution histogram. The scale bar corresponds to 10 mm.

After the target depth and its uncertainty are estimated, we determine the transverse margins of the fluorophore concentration by using Eq. (9). The resulting reconstructions depend on the regularization parameter σ. If σ is too large, the resulting images typically contain significant random noise, which renders the target unrecognizable [see Fig. 4(a)]. On the other hand, if σ is too small, the reconstructed image shows only a blob [see Fig. 4(c)]. To determine the optimum σ, we apply the weak positivity constraint on $C(\rho ,z=z_{\text{target}})$. Since C is intrinsically positive, we require that the mean value of the normalized concentration over all pixels is at least one standard deviation larger than zero; this roughly corresponds to the condition that the fraction of pixels holding negative values is less than 16%. The optimal σ is the maximum σ satisfying the constraint. Note, the regularization parameter, σ, does not impose any assumptions about the spatial variation of fluorophore concentration nor symmetry of the inclusions. The optimized image is shown in Fig. 4(b), and the transverse images of the middle column of Fig. 3 are produced from the same positivity constraint.

**Fig. 5:**
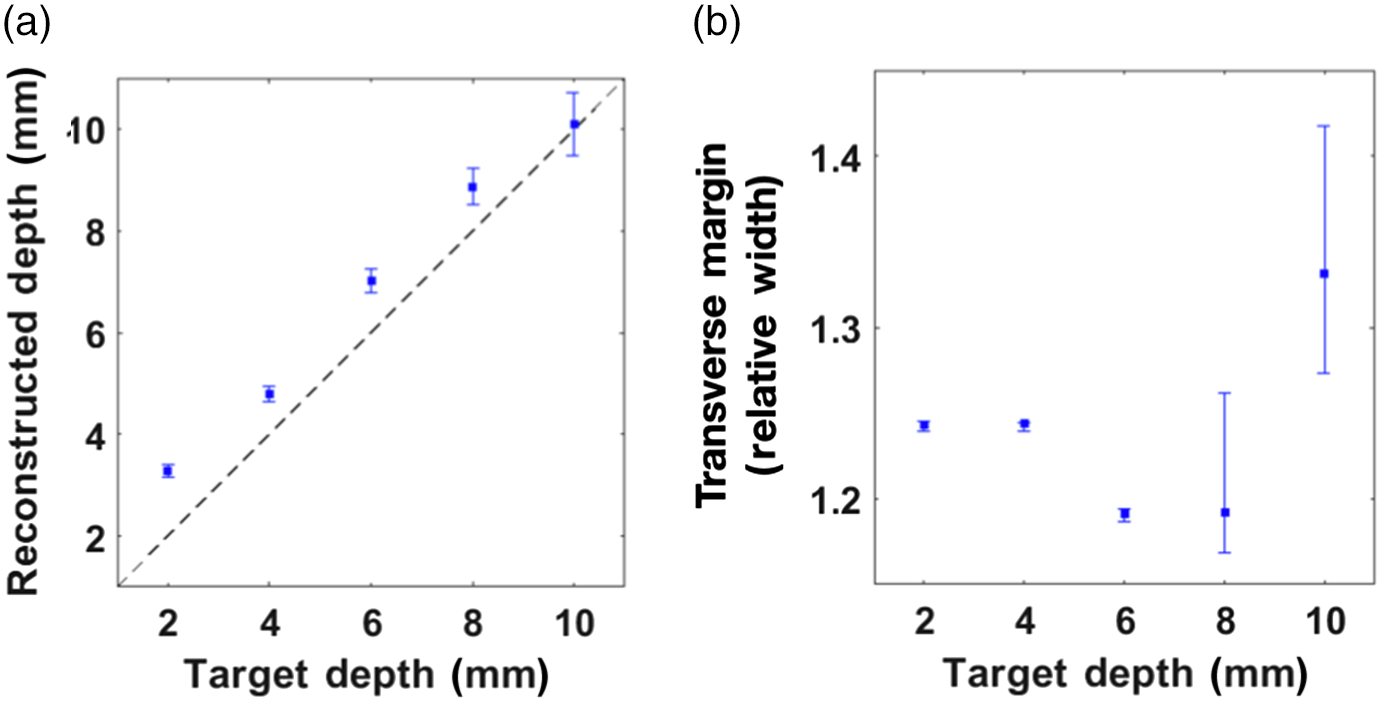
Reconstruction results of single-target experiments. (a) Depth-estimation results. The black dotted line has a unity slope. (b) The ratio between reconstructed image width and the true target width as a function of depth; margin estimate error varies from 20% to 33%.

The lateral margin of the target was set to be the full-width-at-half-maximum (FWHM) contour line of this distribution (the last column of Fig. 3). To measure the discrepancy between the reconstructed and true margins, we computed the relative width—defined as the square root of the ratio of transverse target area (area confined by the blue line in the last column of Fig. 3 to the true target area ($100\;{\mathrm{mm}}^{2}$). For the single-target experiments, the reconstructed depth and the relative width are fairly accurate and are summarized in Fig. 5.

### 3.2 Two-Target Experiments

**Fig. 6:**
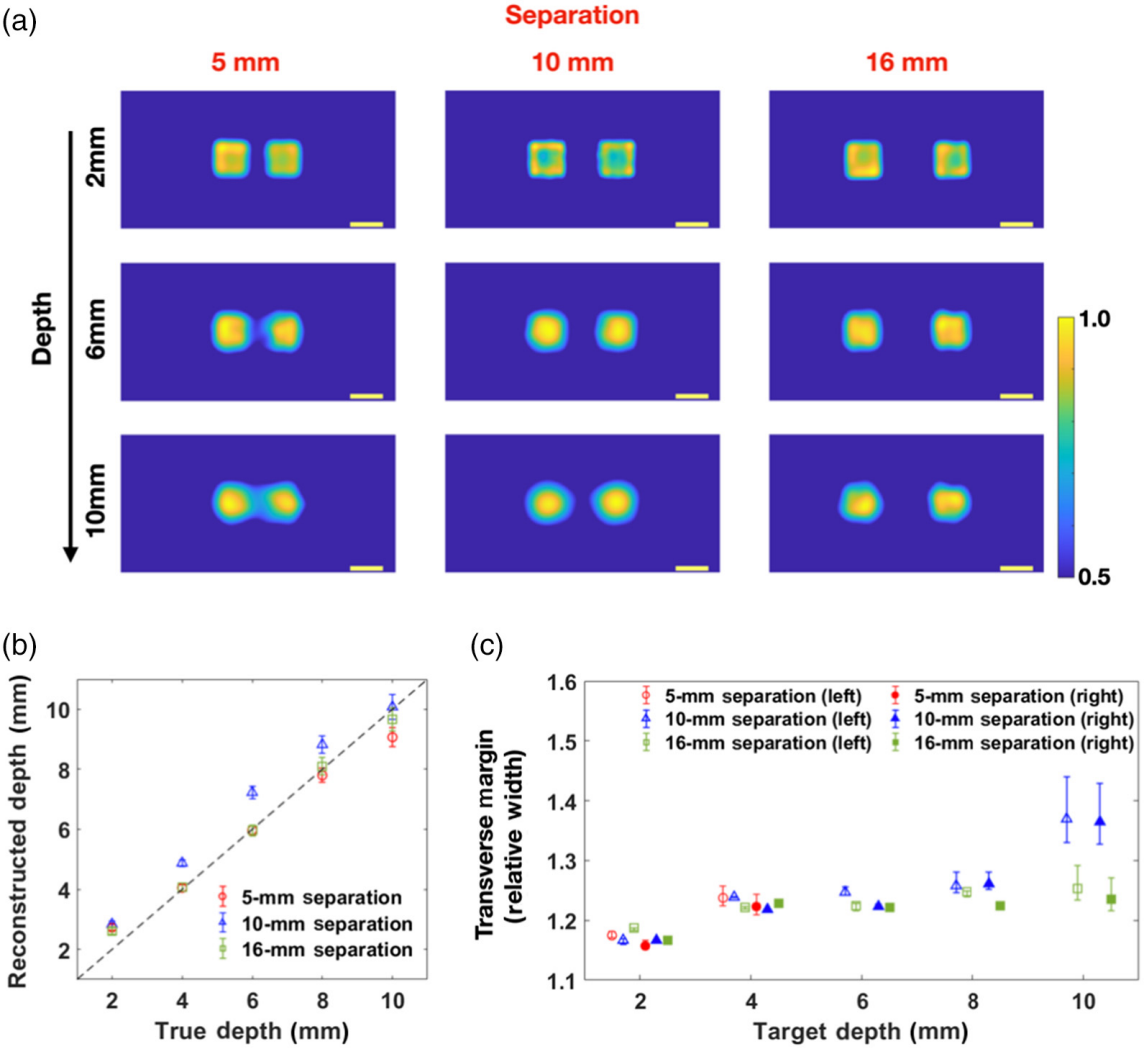
Reconstruction results of two targets in the same vertical plane. (a) Reconstructed transverse margin. When the separation increases, the cross-talk between the targets is decreased. (b) Depth-estimation results. (c) Relative width estimation results. For clarity, we spread the data horizontally so that a “cluster” of points arises at each target depth.

We first placed two identical targets in the same plane but with varying lateral separation. The estimated depths and transverse margins were then computed. A few examples are shown in Fig. 6(a) The two targets are clearly distinguished in most cases. As expected, the ability to distinguish between the targets is reduced as the depth is increased. However, for the range of separations used in our experiments, we clearly observe two targets at every depth. A summary of depth sensitivity and relative width is given in Figs. 6(b) and 6(c).

**Fig. 7:**
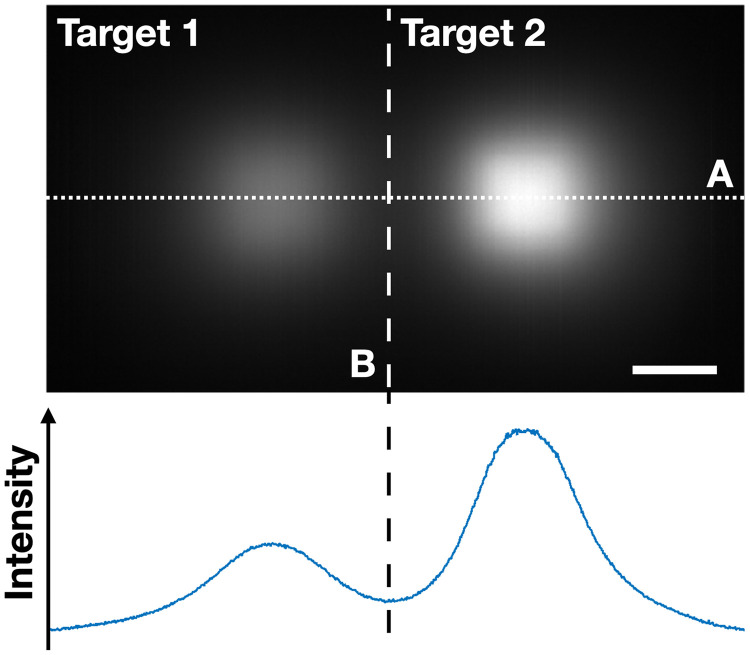
Raw data showing substantially different emission signals from targets 1 and 2. The more deeply located target is target 1, and the more superficial target is target 2. The intensity profile is plotted along the dotted line A. The vertical dashed line indicated by B defines the two segmented regions. The scale bar corresponds to 10 mm.

The second, more challenging problem is to reconstruct two targets that are separated both vertically and laterally. We fixed the lateral separations to be either 20 or 40 mm. The vertical separations were set to be 2, 4, and 6 mm; the depth of Target 2 was fixed at 2 mm below the surface and the depth of Target 1 was varied taking the values 4, 6, and 8 mm. Since the targets were well separated transversely, even when they are located at different depths, we can safely segment the data to process each target separately (Fig. 7). We use the minimum in the peak intensity profile along the horizontal (dotted) line to define the segmentation regions for the image as is shown by the vertical (dashed) lines in figure. After the depth of each target was independently estimated, we used these estimates to process the data further for each target. We obtained the transverse fluorophore distributions by utilizing the same algorithm as in the single target reconstructions.

**Fig. 8:**
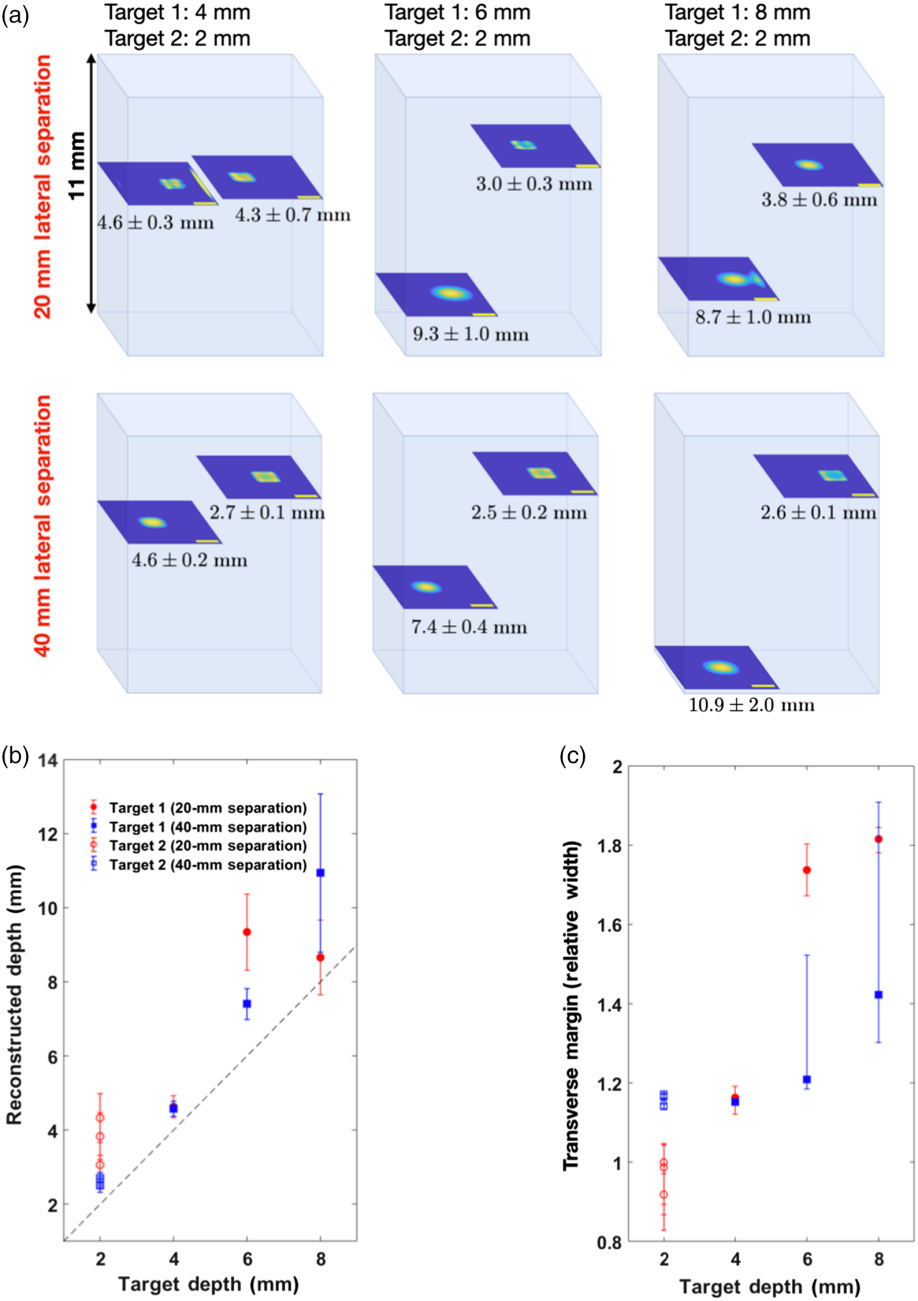
Results of the two-target experiments with vertical separations of the targets. (a) 3D rendering of the results. Columns and rows represent the depth of target 1 and the transverse separation of the targets, respectively (Target 2 is fixed at the 2 mm depth). Estimated depths are marked below each margin slice. The yellow scale bar represents a 10 mm length in the transverse plane. The vertical scale is exaggerated for better visibility. (b) Results for the reconstructed depth of targets 1 and 2. The dashed line has the unity slope. (c) Results of the relative width of targets 1 and 2.

Reconstructions with 20 and 40 mm separations between the targets are presented in Fig. 8(a). The depth-sensitivity and depth estimates for these targets [Fig. 8(b)] are not as good as our results for single targets and for two-targets in the same horizontal plane [Figs. 5(b) and 6(b)]. This can be understood by noting that both the experimental acquisition time and the resulting computationally processed data are different for different target depths. As described in Sec. 2, the camera exposure times needed to obtain reliable fluorescence images for deeper targets are significantly larger than those for shallow targets. In our instrument and data collection scheme, it is not possible to use large exposure times when a shallow target is present due to the possibility of overexposure. We were, therefore, forced to use the exposure times appropriate for the more shallow target. Thus, when target 1 is located at the depth of 10 mm, its fluorescent signal was not detectable due to the insufficient exposure time. Cross-talk of the targets located at different depths can also cause artifacts. As expected, the errors of the transverse margins of the target are larger for smaller horizontal separations [Fig. 8(c)]. When the transverse separation is 20 mm, the cross-talk from target 2 in the segmented image of target 1 becomes significant and produces an artifact near the edge. When this effect is severe, the transverse margins of the deeper target are corrupted [see last column of the first row of Fig. 8(a)]. In the case of the middle column of the first row of Fig. 8(a), the overestimation of the depth is significant. As a result, the two-point Green’s function kernel [Eq. (5)] decays faster as a function of q, which forces strong regularization for transverse margin reconstruction. The strong regularization suppresses the bleeding artifact and produces an image of a nearly featureless large blob.

## 4 Discussion

We have introduced and demonstrated a simple method based on SFD-FDOT to estimate the depth and lateral margins of fluorescent targets in turbid media in the reflection geometry. The work builds upon prior research^19^ in several ways. The targets studied are extended rather than point-like, and they are located as deep as ∼1  cm below the surface rather than within 3 mm. Moreover, we investigated multiple targets located at both the same and different depths, i.e., rather than a single isolated point-like target. A final important and subtle feature of our work, which differs from prior reports, is that we were able to determine the depth of each target without sensitivity to the regularization parameter; this is because the reconstruction was divided into two steps. The first step involves a fitting of the data to a single exponent, and the second step involves a straightforward 2D Fourier transform. In essence, for the second step, we have utilized the prior knowledge that the fluorescence signal is mostly emitted from one particular depth, i.e., from the top surface of the target. Approaches that do not rely on these simplifying assumptions generally require inversion of a severely ill-posed operator. In the latter case, sensitivity to the regularization parameter can and often does become strong.

### 4.1 Depth Sensitivity

**Fig. 9:**
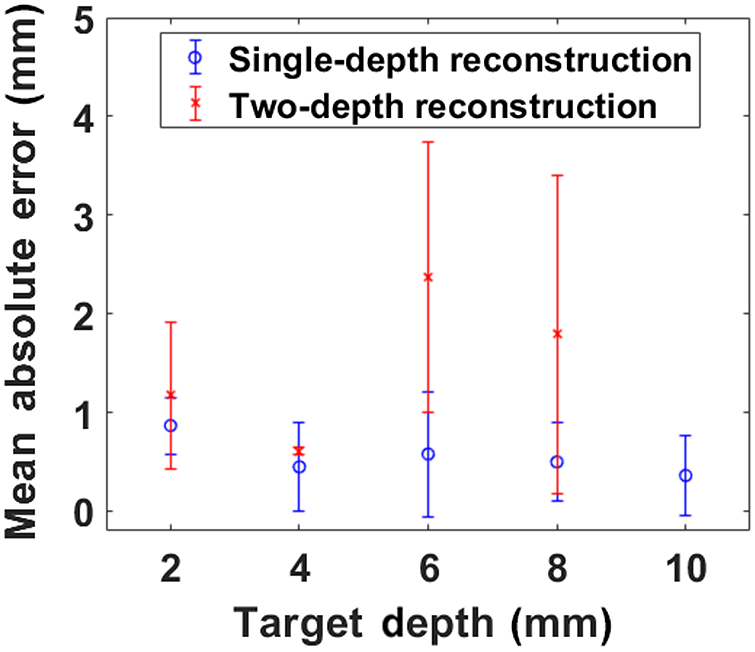
Mean absolute errors for all depth estimation results.

The simple algorithm proposed in this paper successfully estimated the depth of fluorescent inclusions to within ∼1  mm of their true depth in both single target and the two target experiments in which the targets were at the same depth but laterally separated. Prior published work by Konecky et al.^19^ and by Li et al.^24^ also employed the spatial frequency domain method. However, both works used an image reconstruction algorithm based on the pseudoinverse technique. In this algorithm, noise is suppressed by regularization, which often results in a low-contrast image in which the reconstructed fluorophore target stretches further than its true size; for this reason, the accuracy of reconstructed target depth and transverse margins is limited. By contrast, our technique uses a simple exponential relationship between the fluorescence emission signal k-dependence (i.e., dependence on modulation frequency) and the target depth; in this approach, the noise floor [$y_{o}$ in Eq. (8a)] is decoupled from depth information in the exponential decay. Thus, our results are superior to the previous SFD-FDOT simulation results by Li et al.^24^ wherein the accuracy of reconstructed simulated data degraded with increased target depth. (Note, our experimental depth sensitivity results are comparable to simulated data results reconstructed by neural-network-aided FDOT.^27^^,^^29^) Interestingly, our estimation error (both absolute and especially fractional) for single targets was minimum at the largest depth (Fig. 9). The finite target thickness introduces nonlinearities into the reconstruction problem that we do not account for; these effects, briefly discussed in Sec. 3.1, were ameliorated by removing the data point k=0 in the fitting procedure, but it does not fully resolve the effect for all other values of k. However, as the target moves away from the surface the nonlinear effects of target thickness diminish, leading to better depth estimation. In the case of two targets at different depths, the modulation response from more deeply located targets severely suffered cross-talk from the superficially located target. This, and the experimental limitations related to the available exposure times, produced a larger error in terms of depth estimation in this set of experiments, typically, of the order of 20% to 40% of the true depth.

Clearly, some technical improvements are possible in the future work. Theoretically, better accounting for the effect of finite target thickness will potentially allow more precise fitting of depth. This will increase the dynamic range of useful data and reduce the systematic errors of the theoretical model. In addition to reducing depth estimation errors of shallow targets, such improvements can enable imaging of targets located deeper than 10 mm below the surface.

### 4.2 Transverse Margins

**Fig. 10:**
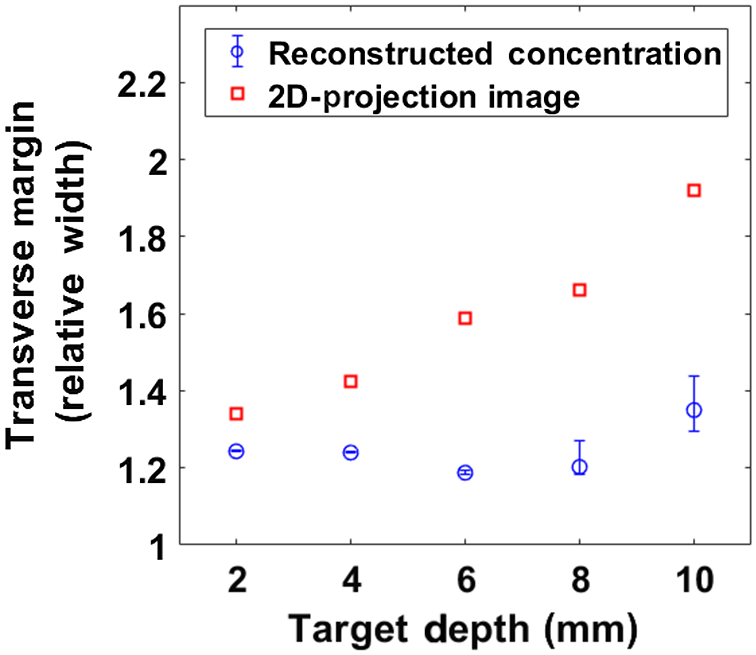
Transverse margin comparison between our reconstruction approach and estimation based on 2D-projection images of fluorescence based on the single-target experiments.

Another important advance is the improved ability to constrain the transverse margins of the fluorescent inclusions (tumors). From the single target experiments, we compared the transverse margin estimates of our reconstructions of fluorophore concentration versus those of traditional 2D fluorescence projection images. (The transverse margins were set to be the FWHM of the image contours.) The relative transverse margin width obtained by each technique is plotted in Fig. 10 as function of target depth. Notice, the traditional 2D fluorescence projection images increasingly overestimate the transverse margins as the target depth becomes larger. For example, at ∼1  cm target depth, the error of the 2D projection image margins was roughly 2.5 times larger than of the margins obtained by our method. The transverse margins of the single targets, or two targets in the same plane, mostly overestimated the true margins by 30% or less. For the two-target experiments with targets at different depth, however, these margin errors were larger, in part because the larger error in depth estimation propagates to the error in transverse margin (Fig. 8(b,c)).

### 4.3 Spatial Smoothing (Regularization) Parameter

**Fig. 11:**
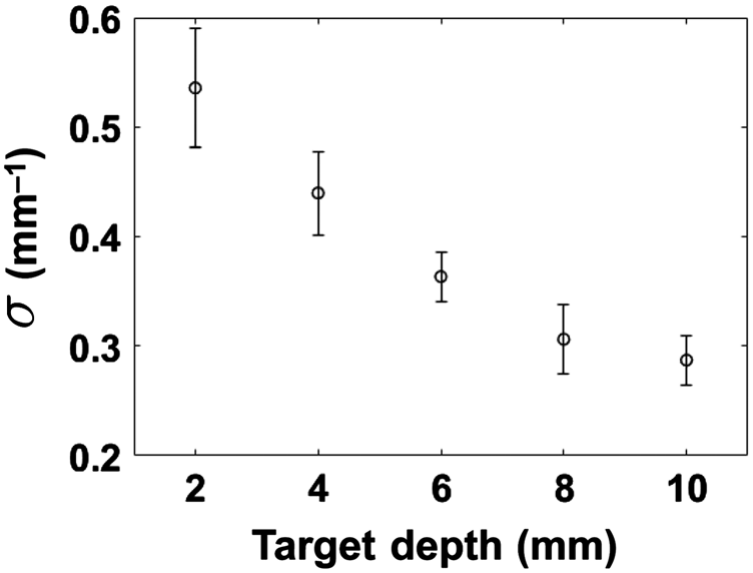
Optimized regularization parameter σ for single-depth reconstruction as a function of the target depth.

Figure 11 shows the average value of the optimized regularization parameter σ as a function of the target depth for single-depth reconstructions. It can be seen that the regularization parameter tends to decrease as the target depth increases. This is because the signal-to-noise ratio for the detected fluorescent intensity decreases as the pathlength of light increases and the high spatial frequencies in the detected intensity distribution become dominated by noise. Empirically, we note that the value of k for which y(k) settles into the noise plateau is roughly proportional to the optimal σ (roughly, k∼σ). Therefore, the value of k settling into the noise plateau can be considered as a guideline for choosing the optimal σ.

### 4.4 Stability of the Estimates

The proposed two-step reconstruction method utilizes Green’s function formalism, which is affected by the optical properties of the background. Therefore, errors in the background optical properties are expected to propagate to errors in estimation of the depth and transverse margin of the fluorescent target. We tested the stability of the reconstructions by introducing a ±10% error into $\mu_{a}$ and $\mu_{s}^{'}$. The resulting absolute error of depth estimation was <0.8  mm, which does not significantly impact the overall depth-sensitivity. Similarly, the change in transverse margins for the targets at the depths of 2, 4, and 6 mm was <2%. Thus, while it is desirable to use the most accurate background optical properties, our method appears to provide fairly stable results for optical property variations of order 10% or less.

### 4.5 Data Acquisition and Reconstruction Times

Data acquisition time introduces the longest temporal delays in our experiments, typically, a few minutes. This time can be reduced. For example, the 31 distinct spatial frequencies currently used are probably more than necessary for good quality imaging. If we remove every second data point from the left panel of Fig. 3, the quality of nonlinear fit is not significantly affected. In this scenario, the image quality is the same, and the data acquisition time is reduced by a factor of two. On the other hand, in some situations additional data points can help improve signal-to-noise. Future work is needed to optimize the number of spatial frequencies for specific instruments and instrumentation parameters.

Since our technique does not attempt to reconstruct full 3D-shapes, the time needed to estimate the depth and transverse margins ranged from 1 to 2 s (Intel(R) Core(TM) i5-8600K CPU 3.60 GHz, 6 Cores). Clearly, the approximate approach is different from the traditional analytic inversion and nonlinear image reconstruction techniques, which require computation times ranging from minutes to hours depending on complexity (i.e., for traditional DOT techniques). Recently, advances based on more complex techniques, such as confocal time-of-flight FDOT and neural network aided FDOT, have demonstrated improvement in reconstruction runtime (from a few milliseconds to seconds) and spatial resolution (millimeter scale). While these advances are excellent and may surpass our reconstruction speed, their clinical suitability have to be evaluated.

### 4.6 Limitations

The proposed SFD-FDOT estimation methodology is promising albeit with the aforementioned limitations. Notably, reconstruction of absolute fluorophore concentration was not pursued, but absolute concentration is not a feature currently employed for image guidance or diagnosis. Per image guidance, the analysis assumed homogeneous background optical properties, semi-infinite geometry, and a thin slab geometry of the fluorescence inclusion. In practice, heterogeneous tissue optical properties in brain or lung tissue could generate errors in estimates of depth and transverse margin. Even though this assumption is commonly used in diffuse optics analysis, more *in vivo* work needs to be done to fully characterize these limitations. Per the semi-infinite geometry, we note that this approximation has proven adequate for many human and animal studies in the diffuse optics field. Therefore, we expect that the proposed technique will be applicable in many open surgery cases for neuro- or thoracic surgery. In principle, this instrumentation can be modified to concurrently measure surface profiles and thereby deduce the magnitude of the deviation from the assumption; this will also provide concrete data that could be employed to modify the current approach perturbatively to include corrections due to surface curvature. Per the thin slab approximation for the fluorescence inclusion, in practice, the high absorption coefficient of ICG will cause the top surface of the target to absorb most of the excitation light propagating downward, and consequently the fluorescence emission will also be dominated by the signal from the top surface. The thin slab approximation is useful, as long as the user realizes that the target depth corresponds to the top surface of the fluorescent region. Note also, even if tumor tissue is not flat on the top surface, the proposed technique will provide a useful estimate of depth that is skewed toward the shallowest part of the tumor.

Finally, it is desirable to make measurements quickly in the operating room. In our current setup, the longest part of the procedure is data acquisition (up to 8 min for the deepest occlusions). In the future, this can be improved by not using the unnecessary spatial modulation frequencies (at high-q) and increasing the excitation light intensity. Notably, the current light intensity on the sample surface was smaller than the ANSI limit by the factor of 3 (see Appendix A of Ref. 34).

## 5 Summary and Outlook

We modified the SFD-FDOT technique for rapid estimation of fluorophore target depth and lateral margin. The methodology was demonstrated to provide depth sensitivity in a variety of experimental situations. The width of the target was estimated with a reasonable negative margin, although this information became less reliable for multiple targets with large vertical separations. These advances build upon the prior work where the target size and depth were limited to point-like occlusions located <3  mm below the surface. Moreover, the prior image reconstructions were sensitive to the regularization parameter and the depth resolution was relatively low. The simple and straightforward analytic estimates proposed in the present work are potentially attractive for the neuro- and thoracic surgeries as the technique can deliver information about the depth and transverse margins of a fluorescing target rapidly and accurately.

More broadly, the fluorophore target information obtained in this simple way can provide priors to constrain more complex fluorescence tomography. Applications in this case, could extend beyond image guidance during the tumor resection surgery. Looking forward, the technique can be improved with more rapid and complete data acquisition and type. Additionally, the use of improved theoretical models accounting for the thickness of the targets can also lead to improvements.

## Code, Data, and Materials Availability

The data used in this paper is available upon request.
